# Efficacy of Direct Renin Inhibitors in Slowing Down the Progression of Diabetic Kidney Disease: A Meta-Analysis

**DOI:** 10.7759/cureus.28608

**Published:** 2022-08-30

**Authors:** Hanieh Akbariromani, Rushna Haseeb, Sumreen Nazly, Sushmita Pandey, Venkata Anirudh Chunchu, Sandesh Dhakal, Mary Anne Claudine Avena, Neelum Ali

**Affiliations:** 1 Medicine, Islamic Azad University, Tehran, IRN; 2 Medicine, Jinnah Hospital Lahore (JHL)/Allama Iqbal Medical College (AIMC), Lahore, PAK; 3 Medicine, University Medical and Dental College Faisalabad, Faisalabad, PAK; 4 Medicine, Maitribodhi Clinic, Kathmandu, NPL; 5 Medicine, Avalon University School of Medicine, Willemstad, CUW; 6 Internal Medicine, College of Medical Sciences, Kathmandu, NPL; 7 Medicine, University of the East Ramon Magsaysay, Quezon City, PHL; 8 Internal Medicine, University of Health Sciences, Lahore, PAK

**Keywords:** progression, albuminuria, meta-analysis, direct renin inhibitor, diabetic kidney disease

## Abstract

Albuminuria is a risk factor for chronic kidney disease and cardiovascular events in diabetic people. The pathogenic processes in these circumstances have been documented to be significantly influenced by enhanced renin-angiotensin system activity. The current meta-analysis was carried out to assess the efficacy of direct renin inhibitors in preventing the progression of diabetic kidney disease. This meta-analysis was conducted as per the guidelines of the Preferred Reporting Items for Systematic Reviews and Meta-Analyses (PRISMA). We searched the relevant medical literature through PubMed, Cochrane library and EMBASE. The primary efficacy outcome was a percentage change in urine albumin-creatinine ratio (UACR) (in mg/g) level. Other primary efficacy outcomes included remission from microalbuminuria to normal albuminuria and progression from microalbuminuria to macroalbuminuria. Four randomized control studies were identified and included in the current meta-analysis involving 9,609 participants. The use of direct renin inhibitors was superior in reducing mean UACR compared to angiotensin receptor blockers and angiotensin-converting enzyme inhibitors. The pooled mean difference in UACR between direct renin inhibitors and the control group was 9.42% (95% CI: -15.70 to -3.15: p-value=0.003). The odds of progression from microalbuminuria to normal albuminuria are 1.26 times higher in patients receiving direct renin inhibitors compared to patients in the control group (OR: 1.26, 95% CI: 1.08-1.46, p-value=0.002). The odds of remission from microalbuminuria to macroalbuminuria were 20% lower in patients receiving direct renin inhibitors compared to patients in the control group (OR: 0.80, 95% CI: 0.69-0.93, p-value=0.003). The use of aliskiren is associated with a significant reduction in UACR, increased remission from microalbuminuria to normal albuminuria and decreased progression from microalbuminuria to macroalbuminuria.

## Introduction and background

Diabetic kidney disease is one of the leading causes of end-stage renal disease (ESRD) in the United States [1] and western countries [2]. Diabetic kidney disease prevalence is 20% to 30% among patients with type 1 diabetes and types 2 diabetes [3]. However, an increased percentage of individuals with type 1 diabetes progressed to ESRD [3]. Diabetic kidney disease is characterized by a progressive decline in estimated glomerular filtration rate (eGFR) and persistent albuminuria. Glomerular hyperfiltration, early kidney disease (microalbuminuria: urine albumin-excretion ratio (UAER) 30 to 300 mg/d), overt kidney disease (macroalbuminuria: UAER > 300 mg/d), and ESRD are the stages of disease that are common to both type 1 and type 2 diabetes [4].

One of the important strategies to prevent the progression of chronic kidney disease in people with diabetic kidney disease is the utilization of renin-angiotensin-aldosterone system (RAAS) inhibitors. Blockage of RAAS can be attained by aldosterone antagonists, angiotensin II receptor blockers, angiotensin-converting enzyme inhibitors and direct renin inhibitors [5]. Blockage of RAAS utilizing angiotensin-converting enzyme inhibitors and angiotensin II receptor blockers has been found to reduce proteinuria, thus slowing diabetic kidney disease progression and reducing cardiovascular mortality and morbidity [6]. Blockage of receptors using angiotensin-converting enzyme inhibitors and angiotensin II receptor blockers alone is incomplete and associated with increased levels of renin and angiotensin that may lead to unsustained, incomplete and weak suppression of aldosterone, thus leading to blunt response [7].

The direct renin inhibitors now sold commercially are imarikiren and aliskiren. Aliskiren is responsible for increased renal vasodilation showing better blockages of RAAS compared to angiotensin-converting enzyme inhibitors and angiotensin II receptor blockers in healthy individuals [8]. These effects might be particularly noticeable in diabetics with activated RAAS systems. Direct renin inhibitors seem to provide more efficient RAAS blockade by preventing the escape of aldosterone in combination with angiotensin II receptor blockers or angiotensin-converting enzyme inhibitors therapy. The study conducted by Parving et al. found that aliskiren significantly reduced proteinuria when given with angiotensin II receptor blockers independent of its blood pressure-lowering effects [9]. In addition, the total number of serious adverse events and adverse events remained similar in both the placebo and aliskiren groups [9].

As far as our knowledge goes, this is the first meta-analysis that compared the efficacy of direct renin inhibitors alone or in combination with angiotensin II receptor blockers or angiotensin-converting enzyme inhibitors therapy. During the last 20 years, the outlook for diabetes patients with diabetic kidney disease has improved, probably because of the early aggressive lowering of blood pressure and blocking of the renin-angiotensin-aldosterone system [10]. However, still, a large, unmet need is there to form strategies for the prevention of the progression of chronic kidney disease. Thus, the current meta-analysis was carried out to assess the efficacy of direct renin inhibitors in the prevention of the progression of diabetic kidney disease.

## Review

Methods

This meta-analysis was conducted as per the guidelines of the Preferred Reporting Items for Systematic Reviews and Meta-Analyses (PRISMA).

Search Strategy

We searched the relevant medical literature through PubMed, Cochrane library and EMBASE using the following keywords: diabetic kidney disease, diabetic nephropathy, direct renin inhibitors, progression, efficacy, and clinical trial in all languages without putting a restriction on the date of publication. We also reviewed the reference list of all the identified articles to locate further eligible studies. The search strategy described was used to search for relevant articles to determine which studies satisfy the inclusion criteria. Titles and abstracts were screened independently by two authors followed by a full-text screening of relevant articles.

Eligibility Criteria

We included all randomized control trials looking at individuals with diabetic kidney disease receiving direct renin inhibitors either alone or in combination with angiotensin receptor blockers or angiotensin-converting enzyme (ACE) inhibitors that provided data on any of the outcomes assessed in the current meta-analysis (urine albumin-creatinine rate, remission from microalbuminuria to normal albuminuria, progression from microalbuminuria to macroalbuminuria and all-cause mortality) relative to monotherapy with an angiotensin receptor blocker, an ACE inhibitor or placebo. Individuals with diabetic kidney disease (type 1 diabetes and type 2 diabetes) are defined by the presence of urine albumin-creatinine ratio (UACR) > 30 mg/g or UAER > 30 mg/d. We excluded studies carried out in non-diabetic kidney disease patients. We also excluded reviews, observational studies, case series, letters, editorials and commentaries from the current meta-analysis.

Assessment of Risk of Bias

Two reviewers assessed the risk of bias independently of all included studies by using the checklist of the Cochrane Database of Systematic Reviews. The checklist assessed the risk of bias using six domains that included sequence generation, allocation concealment, attrition, blinding, selection bias and other reporting biases. The classification in each category was unclear, yes or no. Discrepancies between two reviewers were resolved by consensus or involvement of the third reviewer. RevMan software (version 5.4.1; Cochrane, London, United Kingdom) was used to draw the risk of bias graph.

Outcomes Measures

The primary efficacy outcome was a percentage change in UACR (in mg/g) level. The percentage changes of values were computed by subtracting the mean of the endpoints from the baseline mean and dividing it by the baseline value. The standard deviation calculation method was adopted from Cochrane Handbook version 5.1.1. Original data were collected in the form of mean and standard deviation. Articles in which median and interquartile ranges were reported and mean and standard deviation were calculated manually using values of the median, interquartile ranges and sample size were collected. Other primary efficacy outcomes included remission from microalbuminuria to normal albuminuria and progression from microalbuminuria to macroalbuminuria. Microalbuminuria is UACR=30-300 mg/g, and macroalbuminuria is UACR >300 mg/g. Secondary efficacy outcomes included changes in estimated glomerular filtration rate in milliliters per minute (mL/min).

Data Extraction

Data were extracted by two reviewers independently using custom-made data extraction forms on Microsoft Excel (Microsoft, Washington, United States). Data extracted included first author name, year of publication, intervention, sample size, follow-up period and outcomes. The original data were not modified, and calculations were carried out from the available data for the current meta-analysis.

Data Synthesis

For data analysis, RevMan software (version 5.4.1) was used. For binary outcomes, risk ratios along with 95% confidence intervals were calculated using Mantel-Haenszel random effect model. For continuous variables, mean differences and their 95% confidence interval were calculated. I^2^ value was used to assess heterogeneity between study results, and the Cochrane Q test (significance set at 0.01) was used for statistical testing of heterogeneity. We were able to assess publication bias because of a limited number of studies.

Results

**Figure 1:**
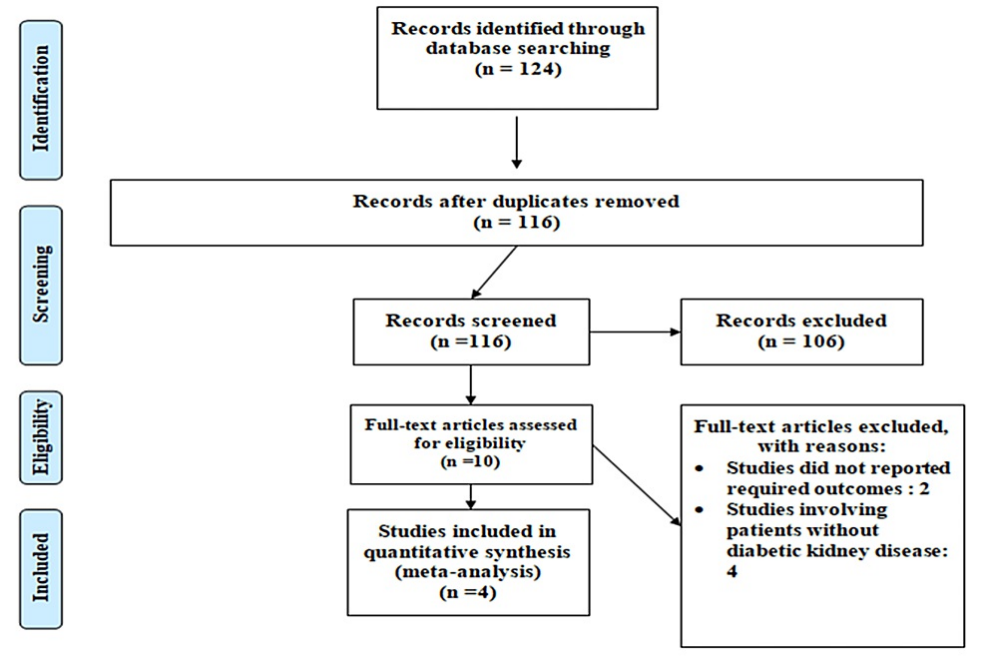
PRISMA flowchart of selection of studies PRISMA: Preferred Reporting Items for Systematic Reviews and Meta-Analyses.

Figure 1 shows the PRISMA flowchart for the selection of studies. Overall, 124 articles were identified through online database searching. After removing duplicates, 116 articles were eligible for the title and abstract screening. Out of 116 articles, a full-text screening of 10 articles was carried out. Among all these studies, four randomized control studies were identified and included in the current meta-analysis involving 9,609 participants. Table 1 shows the characteristics of the included studies and participants.

**Table 1:** Characteristics of the included studies *Mean (standard deviation). GFR: glomerular filtration rate; ARB: angiotensin receptor blocker; ACEI: angiotensin-converting enzyme inhibitors.

Author name	Year	Setting	Intervention	Dose	Sample size	Follow-up	Mean age*	Male (%)	Mean baseline estimated GFR (ml/min)*
Ito et al. [11]	2019	Multicenter	Imarikiren	5, 20, 40 and 80 mg/day	277	14 weeks	61 (10)	219 (79.06)	80 (19)
			ARB		70		62 (9)	56 (80)	83 (23)
Parving et al. [9]	2008	Multicenter	Aliskiren+ARB	150 mg/day	301	24 weeks	59.9 (9.6)	206 (68.4)	68.5 (25.7)
			ARB		298		61.8 (9.6)	221 (74.2)	66.8 (24.5)
Parving et al. [12]	2012	Multicenter	Aliskiren+ARB/ACEI	300 mg/day	4,274	78 weeks	64.6 (9.6)	2,881 (67.4)	57.0 (21.9)
			ARB/ACEI		4,287		64.4 (9.9)	2,945 (68.7)	57.0 (23.0)
Uzu et al. [13]	2016	Multicenter	Aliskiren	150 mg/day	49	24 weeks	64.3 (10.2)	36 (73.0)	79.2 (24.3)
			ARB		53		63.3 (8.0)	37 (70.0)	73.9 (2.1)

Of all the included studies, two compared the monotherapy of direct renin inhibitors with angiotensin receptor blocker (ARB) [11,13], while two studies compared the combination of direct renin inhibitors with monotherapy of ARB or angiotensin-converting enzyme inhibitor (ACEI) [9,12]. The dose for ARB, ACEI and direct renin inhibitors varied in the included study. However, all included randomized controlled trials (RCTs) were designed to reach the maximum dose for direct renin inhibitors. The duration of the study ranged from 14 weeks to 78 weeks. Participants were mostly male and relatively older.

Assessment of Risk of Bias

**Figure 2:**
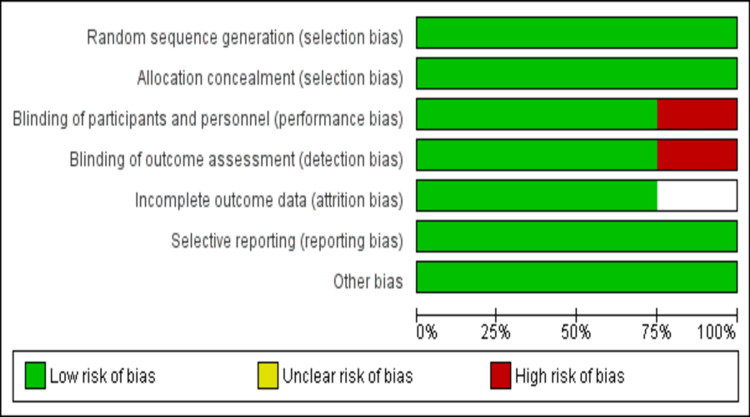
Risk of bias graph

The overall risk of bias was moderate. All four studies discussed the methods used for the generation of the randomization sequences and details related to allocation concealment. Three studies reported blinding of participants and investigators, and one RCT was open-label. All efficacy outcomes were based on the laboratory data; this will less likely affect these outcomes. The withdrawal rate of all studies was less than 20%. Figure 2 shows the risk of bias assessment graph.

Primary Efficacy Outcomes

**Figure 3:**
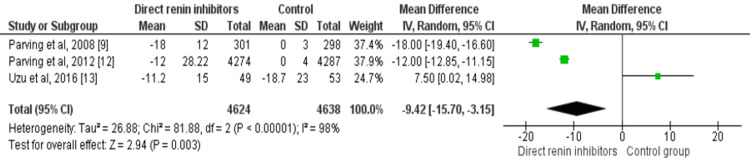
Forest plot on the impact of direct renin inhibitors on percentage change in UACR Sources: References [9,12-13]. UACR: urine albumin-creatinine ratio; df: degree of freedom.

Three out of four included studies assessed the impact of direct renin inhibitors on UACR involving overall 9,262 patients with type 2 diabetes. The use of direct renin inhibitors was superior in reducing mean UACR. The pooled mean difference in UACR was -9.42% (95% CI: -15.70 to -3.15: p-value=0.003). There was a significant heterogeneity (p-value<0.001) as shown in Figure 3.

**Figure 4:**
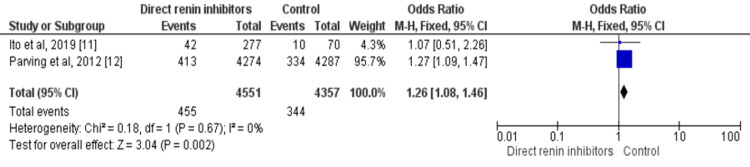
Forest plot on the effect of direct renin inhibitors on remission from microalbuminuria to normal albuminuria Sources: References [11-12]. df: degree of freedom, M-H: Mantel-Haenszel method.

Two out of four studies assessed the impact of direct renin inhibitors on remission from microalbuminuria to normal albuminuria including a total of 8,904 patients. Among patients who received direct renin inhibitors, 10% of patients progressed from microalbuminuria to normal albuminuria compared to 7.89% of patients in the control group. The odds of progression from microalbuminuria to normal albuminuria are 1.26 times higher in patients receiving direct renin inhibitors compared to the control group (OR: 1.26, 95% CI: 1.08-1.46, p-value=0.002). No significant heterogeneity was found among the study results (p-value=0.67) as shown in Figure 4.

**Figure 5:**
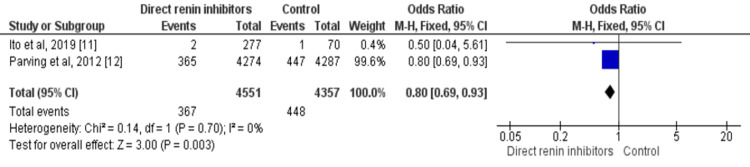
Forest plot on the impact of direct renin inhibitors on progression from microalbuminuria to macroalbuminuria Sources: References [11-12]. df: degree of freedom, M-H: Mantel-Haenszel method.

Two out of four studies assessed the impact of direct renin inhibitors on progression from microalbuminuria to macroalbuminuria including a total of 8,904 patients with diabetic kidney disease. The odds of macroalbuminuria are 20% lower in patients receiving direct renin inhibitors compared to patients in the control group (OR: 0.80, 95% CI: 0.69-0.93, p-value=0.003). Heterogeneity was insignificant among the study results (p-value=0.70) as shown in Figure 5.

Secondary Efficacy Outcomes

**Figure 6:**
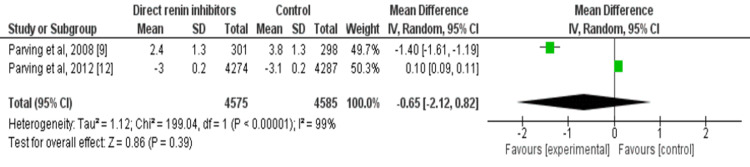
Forest plot on the impact of direct renin inhibitors on GFR Sources: References [9,12]. GFR: glomerular filtration rate; df: degree of freedom.

Two out of four studies assessed the impact of direct renin inhibitors on eGFR including overall 9,160 patients with type 2 diabetes. The direct renin inhibitor was not better than the control group in reducing eGFR in diabetes patients. The pooled mean difference in eGFR was -0.65 (95% CI: -2.12 to 0.82, p=0.39) as shown in Figure 6.

Safety Outcome

**Figure 7:**
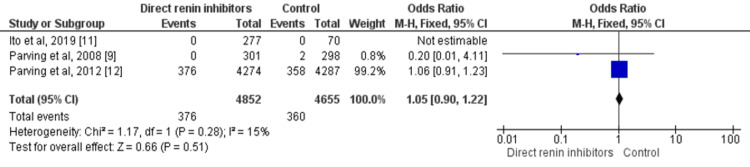
Forest plot on the impact of direct renin inhibitors on all-cause mortality Sources: References [9,11-12]. df: degree of freedom, M-H: Mantel-Haenszel method.

Three studies assessed all-cause mortality including 9,507 patients with type 2 diabetes. No significant differences were reported in all-cause mortality between the two groups (OR: 0.93, 95% CI: 0.39-2.24, p-value=0.87) as shown in Figure 7.

Sensitivity Analysis

A sensitivity analysis was performed by comparing the results of direct renin inhibitors as monotherapy with a combination of direct renin inhibitors and ACEI or ARB. Table 2 shows the results of the sensitivity analysis. There is a significant reduction of UACR in combination therapy as compared to ARB alone. However, when direct renin inhibitors were given alone, an increase in percentage change in UACR was observed. In addition, when direct renin inhibitors were given in combination with ARB or ACEI, the odds of remission from microalbuminuria to normal albuminuria are significantly higher compared to ARB or ACEI only. However, monotherapy of direct renin inhibitors did not significantly improve remission. On the other hand, progression from microalbuminuria to macroalbuminuria is significantly lower in patients who received the combination of drugs.

**Table 2:** Results of sensitivity analysis *Significant at p-value<0.05. All values are presented as OR (95% CI) except UACR that is presented as mean percentage change with 95% CI. Combination therapy: direct renin inhibitor+angiotensin receptor blockers or angiotensin-converting-enzyme inhibitors. UACR: urine albumin-creatinine ratio.

Outcomes	Direct renin inhibitors	
	Monotherapy	Combination
UACR	7.50 (0.02, 14.98)*	-14.97 (-20.85, -9.09)*
Remission from microalbuminuria to normal albuminuria	1.07 (0.51-2.26)	1.27 (1.09-1.47)*
Progression from microalbuminuria to macroalbuminuria	0.50 (0.04-5.61)	0.80 (0.69-0.93)*

Discussion

It is well recognized that one of the most effective therapeutic approaches for lowering the risk of cardiovascular and renal events in diabetic patients is to treat microalbuminuria by blocking the renin-angiotensin-aldosterone pathway [13]. In the present meta-analysis, direct renin inhibitor was associated with a significant reduction in UACR and also improvement in other outcomes including remission from microalbuminuria to normal albuminuria and progression from microalbuminuria to macroalbuminuria. To date, no published meta-analysis and systematic review have been conducted to evaluate the efficacy of direct renin inhibitors as monotherapy or in combination with ARB and ACEI.

Since the hazard of end-stage kidney disease is reported to be associated with albuminuria in past observational studies [14-15], a decrease in albuminuria is usually linked to a reduced risk of end-stage kidney disease. However, previous findings suggested that albuminuria might be a poor surrogate. It means that a decrease in the level of albuminuria often translates into long-term renoprotection could be wrong [16]. There has been much debate on whether albuminuria is a reliable surrogate for other outcomes. The effectiveness of direct renin inhibitors to enhance remission from microalbuminuria to normal albuminuria is evident in the current meta-analysis. However, in the current meta-analysis, combination therapy of aliskiren with either ARB or ACEI was found to be better in controlling the level of albuminuria. The enhancement in remission rates is notable considering that normalization of albuminuria has been associated with enhanced cardiovascular and renal outcomes in diabetic kidney patients [17].

Aliskiren is an efficient antihypertensive agent, and when it is added to either an ARB or ACEI, the enhanced surrogate markers for outcomes in certain studies are not specific and seem to suggest additional benefits that have not been demonstrated. For instance, it reduced urinary albumin excretion in diabetic kidney disease patients [18]. In this case, the findings of the Aliskiren in the Evaluation of Proteinuria in Diabetes trial, which compared aliskiren with placebo in patients with diabetic renal impairment who were also receiving losartan medication, are very significant [8]. In one study, patients who achieved a 50% decrease in UACR had a 59% lower adjusted hazard for kidney and cardiovascular events as compared to those individuals without a 50% reduction [19].

Regarding the safety of direct renin inhibitors, no significant difference was reported in terms of all-cause mortality. However, a past meta-analysis conducted on patients with nondiabetic kidney disease has raised issues on adverse events. The meta-analysis showed that combination therapy of direct renin inhibitors was associated with an increased risk of moderate hyperkalemia [20]. Prior studies had established the higher risks of hypotension and hyperkalemia associated with the combination of an ACE inhibitor and an ARB [21-22]. The randomized control trial conducted by Parving et al. reported more severe outcomes in patients receiving combination therapy of aliskiren and ARB [9]. Major adverse events that were significantly higher in the direct renin inhibitor group were hyperkalemia and hypotension. However, studies conducted by Ito et al. [11] and Parving et al. [9] did not show any significant difference between the two groups in terms of adverse events. Considering the benefits of direct renin inhibitors in terms of reducing UACR and albuminuria levels, further studies need to be continued in people with diabetic kidney disease. More surrogate markers of disease progression should be studied to give the best possible treatment to these patients.

Currently, sodium/glucose cotransporter-2 inhibitors (SGLT2i) and finerenone combination therapy are beneficial options in slowing down the progression of diabetic kidney disease. However, direct renin inhibitors can also be used in combination with ARB/ACEI. Future trials and analyses will shed further light on this to determine the benefits and harms of both of these treatment options. The current meta-analysis has certain limitations. Firstly, we were able to include only four randomized control trials as most of the studies were conducted on nondiabetic kidney disease. Due to the limitations of studies, publication bias was also not assessed. Many of these included studies were small, resulting in few adverse safety events, and no significant differences were reported between the groups. That is why we did not compare serious adverse events between the two groups. Studies need to be conducted in the future to assess all measures of renal disease, especially in a high-risk population such as patients with diabetes.

## Conclusions

The use of aliskiren is associated with a significant reduction in UACR, increased remission from microalbuminuria to normal albuminuria and decreased progression from microalbuminuria to macroalbuminuria. It is also evident in the current meta-analysis that combination therapy of direct renin inhibitors and ARB is more effective in decreasing the progression of kidney disease among patients with diabetes. Further research to clarify the role and safety of using aliskiren in combination therapy on important clinical outcomes is needed.
